# Drug Delivery Applications of Core-Sheath Nanofibers Prepared by Coaxial Electrospinning: A Review

**DOI:** 10.3390/pharmaceutics11070305

**Published:** 2019-07-01

**Authors:** Bishweshwar Pant, Mira Park, Soo-Jin Park

**Affiliations:** 1 Department of Chemistry, Inha University, 100 Inharo, Incheon 402-751, Korea; 2 Department of Bioenvironmental Chemistry, College of Agriculture & Life Science, Chonbuk National University, Jeonju 561-756, Korea

**Keywords:** electrospinning, coaxial spinning, core-sheath nanofibers, biomedical, drug delivery

## Abstract

Electrospinning has emerged as one of the potential techniques for producing nanofibers. The use of electrospun nanofibers in drug delivery has increased rapidly over recent years due to their valuable properties, which include a large surface area, high porosity, small pore size, superior mechanical properties, and ease of surface modification. A drug loaded nanofiber membrane can be prepared via electrospinning using a model drug and polymer solution; however, the release of the drug from the nanofiber membrane in a safe and controlled way is challenging as a result of the initial burst release. Employing a core-sheath design provides a promising solution for controlling the initial burst release. Numerous studies have reported on the preparation of core-sheath nanofibers by coaxial electrospinning for drug delivery applications. This paper summarizes the physical phenomena, the effects of various parameters in coaxial electrospinning, and the usefulness of core-sheath nanofibers in drug delivery. Furthermore, this report also highlights the future challenges involved in utilizing core-sheath nanofibers for drug delivery applications.

## 1. Electrospinning

To date, several approaches, such as phase separation, self-assembly, drawing, template synthesis, and electrospinning, have been put forward for the fabrication of one-dimensional (1D) fibrous structures from various polymers [1,2,3,4,5,6]. In comparison with other methods, electrospinning is considered to be a simple, versatile, and cost-effective technique for generating continuous, nonwoven nanofiber mats from various polymeric solutions [2,7]. A variety of precursor solutions, including natural and synthetic polymers, polymer blends, polymer/metal particles, and polymer/ceramics particles, have been electrospun into fiber/nanofiber structures with diameters ranging from the nanometer to micrometer scale [1,8]. The electrospun nanofibers bear several remarkable properties such as a small diameter, large surface area, high aspect ratio, unique physiochemical properties, and flexibility [2,9,10].

**Figure 1:**
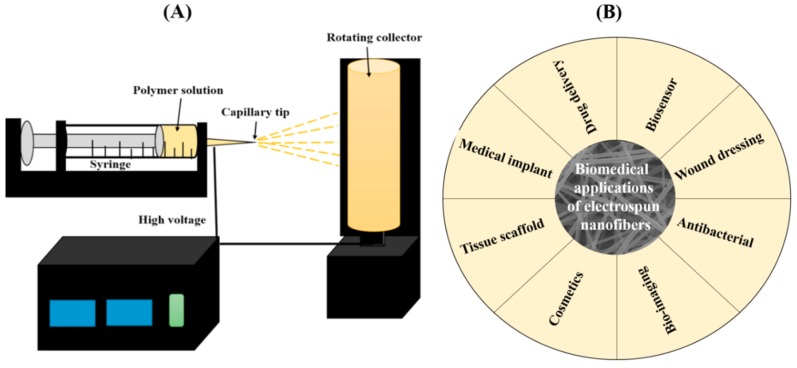
(**A**) Schematic diagram showing the electrospinning setup and (**B**) various biomedical applications of electrospun nanofibers.

The standard electrospinning setup consists of four main components: a capillary tube containing a polymer solution (or melt), a spinneret or nozzle, a collector, and a high voltage source [2,11]. The nanofibers can be obtained either from polymer melt or solution. A majority of the work has been focused on solution-based electrospinning. In the electrospinning process, a high voltage (typically 10 to 30 kV) is applied to a polymer solution in order to induce a charge on the surface of the droplet. When the intensity of the electric field is increased, the hemispherical surface of the solution elongates at the tip of the capillary and forms a Taylor cone. Upon further increasing the applied voltage, the charged jet is ejected from the Taylor cone and flows in the direction of the collector. During this process, the solvent evaporates and dry polymer fibers are randomly deposited on the collector. In electrospinning, the spinneret is the most important component, by which the multiple configurations can be implemented. Depending on the spinneret, the electrospinning process can be divided into different types such as needle-less, single, coaxial, side-by-side, and tri-axial electrospinning. The schematic of the typical electrospinning process is given in Figure 1A.

In the electrospinning process, there are several factors that contribute to the fiber morphology [4,12]. These factors can be divided into tree category: (i) Solution parameters; (ii) process parameters; and (iii) ambient parameters [13]. The solution parameters include the type of solvent, the molecular weight of the polymer, the concentration of the solution, the viscosity of the solution, the conductivity of the solution, the surface tension, the dipole moment, and the dielectric constant. The process parameters include the applied electric field, the distance from the tip of the needle to the collector, the flow rate, etc. The relative humidity and temperature are considered as environmental factors affecting the electrospinning process. All the parameters affect the morphology of the nanofibers. It is noteworthy to mention that all the parameters affect the morphology of the nanofibers and none of them act independently during electrospinning. Therefore, the optimization of the different parameters is essential in order to design a nanofiber with the desired structure and properties. The effect of various parameters on the properties of the electrospun nanofibers is summarized in Table 1.

## 2. Biomedical Applications of the Electrospun Nanofibers

A wide range of polymers have been electrospun to prepare nanofibers. So far, more than 200 polymers have been reported to have been made into nanofiber structures with the electrospinning technique [14]. Electrospun nanofibers possess several outstanding characteristics, for example, a large surface area to volume ratio, superior mechanical strength, flexibility, ease of surface modification, etc., which are beneficial for diverse applications including those in the biomedical sector, energy storage, and environmental remediation [1,4,20,21].

Electrospun nanofibers have already been proposed for various biomedical applications. The structure and chemical composition of the electrospun nanofibers resemble the natural fibrillary extracellular matrix (ECM) [22,23]. The interconnecting porous nature of the electrospun fibers facilitates cells attaching, migrating, and proliferating [23]. The biocompatibility and biodegradability of the nanofibers along with their suitable mechanical properties also favor their use in biomedical applications. Some potential areas of applications are given in Figure 1B. The electrospun nanofibers have been applied in various biomedical applications such as tissue engineering, wound dressing, the biosensor field, drug delivery, stent coating, implants, cosmetics, facial masks, etc. [24,25,26]. Many biocompatible polymers have been electrospun to generate nanofibers to be applied in the biomedical field; these polymers can either be biodegradable or non-biodegradable. Natural polymers such as collagen, chitosan, gelatin, hyaluronic acid, and silk fibroin have been electrospun into nanofiber form to form potential scaffolds for biomedical applications [27,28]. Other polymers include polycaprolactone (PCL), poly (lactic-co-glycolic acid) (PLGA), polylactic acid (PLA), poly (glycolic acid) (PGA), and poly(L-lactide-co-caprolactone) (PLCL) [27,29,30]. Currently, research is focused on three main issues: (i) Scaffold for tissue engineering; (ii) drug delivery mechanisms; and (iii) enzyme immobilization for faster reaction rates in biological reactions.

Among the various applications in the biomedical field, electrospun nanofibers use is potentially very promising in drug delivery applications. The electrospun nanofibers have been used in drug delivery applications for treating various diseases and gained popularity in the field of pharmaceutics [6,30,31,32]. The advantageous characteristics of nanofibers such as high surface area, high drug loading capacity, porosity, ease of functionalization and surface modification, simultaneous delivery of diverse therapies, adequate mechanical strength, and cost-effectiveness are appealing for use in drug delivery systems. Since the Kenaway group [33] prepared tetracycline loaded electrospun nanofiber mats of PLA, poly (ethylene-co-vinyl acetate) (PEVA), and their blend and studied the release of tetracycline for the first time, electrospun nanofiber for drug delivery gained significant interest among researchers. In the past few years, several research groups have prepared polymeric nanofibers in order to achieve different controlled drug release profiles. To date, various types of drugs, including DNA, RNA, protein, antibacterial, antiviral, and anticancer agents, etc., have been incorporated into electrospun nanofibers for desired applications [31,34,35,36]. For example, electrospun nanofibers have been applied as skin care (or facial) masks for the treatment of the skin as well as for other medical or therapeutic purposes. Different types of skin revitalizing factors can be loaded into the electrospun nanofibers and can be applied directly to the skin [37]. In recent years, drug incorporated biocompatible nanofiber membranes have been explored as a stent coating material to store and elute pharmaceutical agents to the lesion site without compromising its functional behavior [38].

## 3. Core-Sheath Nanofibers

**Figure 2:**
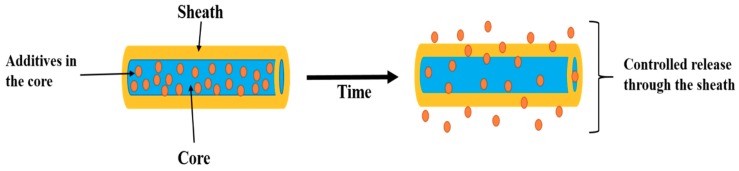
Schematic view showing the encapsulation and release procedure [50].

The main objective of the drug delivery system is to deliver a required amount of a certain drug for a defined period of time depending on the medical condition. A drug loaded nanofiber membrane can be prepared via electrospinning by using a model drug and polymer solution; however, the initial burst release is unavoidable in such a type of blend membrane, and this is not ideal for a sustained drug release. In order to eliminate the burst release, post-treatment of the membranes such as cross-linking or chemical modifications are required. These types of post-treatment may lead to toxicity and a reduction in biocompatibility. The incorporation of the bioactive molecules or drugs into the thin fiber structure remains challenging since it should not adversely affect either the scaffold’s properties or the drug’s activity. The drug release rate can be tailored by tuning fiber diameter, porosity, and the drug-binding mechanism [39,40,41,42,43]. In recent years, many modifications have been incorporated into the electrospinning technique for producing nanofibers with enhanced performances. One such modification is the preparation of core-sheath nanofibers using coaxial electrospinning, in which one polymer nanofiber is surrounded by another, thus benefiting from the properties of both polymers [44,45,46]. In drug delivery systems, coating the fiber with a shell could effectively control the release profile of the drugs [47]. The specific core-shell design is helpful to incorporate the active drugs into the core part of the nanofibers, thereby providing the possibility of avoiding any damage caused to the incorporated drugs. In recent years, several core-sheath nanofibers have been fabricated to load various bioactive molecules including drugs, proteins, and genes for the sustained release of these molecules. The schematic view of the encapsulation and release procedure of drugs in core-sheath nanofibers is given in Figure 2. The advantages of core-sheath nanofibers in drug delivery applications can be summarized as follows [46,47,48,49]:

(i) It is possible to prepare nanofibers from unspinnable solutions via coaxial spinning;
(ii) It is helpful to prevent the burst release;
(iii) It enables a sustained release for a longer time;
(iv) The release kinetics of the bioactive molecules can be controlled by changing the composition or feed rate;
(v) More than one drug can be loaded in the same nanofibers and the drug release rate can be regulated;
(vi) Encapsulating the unstable bioactive molecules in mild conditions and protecting the biological activity of these molecules;
(vii) The sheath layer protects the inner ingredients, governing the release kinetics of the core which contains molecules;
(viii) It provides a better therapeutic effect and reduced toxicity;
(ix) This process eliminates the potential harm that can be caused by the post-treatment process.

### 3.1. Core-Sheath Fibers from Co-Axial Electrospinning

Coaxial or triaxial electrospinning is considered to be an effective strategy to achieve sustained drug release from electrospun core-sheath nanofibers. It involves a simultaneous flow of a core and sheath solution from separate capillaries to form a fiber [51]. Coaxial electrospinning can be used to prepare core-sheath structured fibers from various core and sheath solutions to generate a fiber with different inner and outer parts: a hollow fiber, and functional fibers that may contain coatings [52]. Recently, fabrication of core-sheath fibers with triaxial electrospinning has been introduced in which the electrospun fiber contains a core, middle layer, and sheath [53,54]. By applying coaxial electrospinning, multiple drugs can be loaded into the core-sheath fibers and their release kinetics can be controlled [55]. In comparison to blend spinning, the drug loading efficiency is higher in coaxial spinning. More importantly, the initial burst release is found to be lower in core-sheath nanofibers made by coaxial spinning than that of fibers made by blend spinning. In core-sheath nanofibers, the core swells or dissolves, forming pores in the shell after the dissolutioin of hydrophilic portion in the core. While loading drugs into the core phase, the shell phase can serve as an outer protective layer. Furthermore, incompatibilities can be overcome. For instance, hydrophilic drugs in the core phase can be incorporated into the hydrophobic polymers in the shell phase. Furthermore, the shell phase can serve as a physical barrier providing sustained release kinetics, and by loading the core and shell phase, two different release patterns from one delivery system can be achieved.

### 3.2. General Setup and the Process of Coaxial Electrospinning

**Figure 3:**
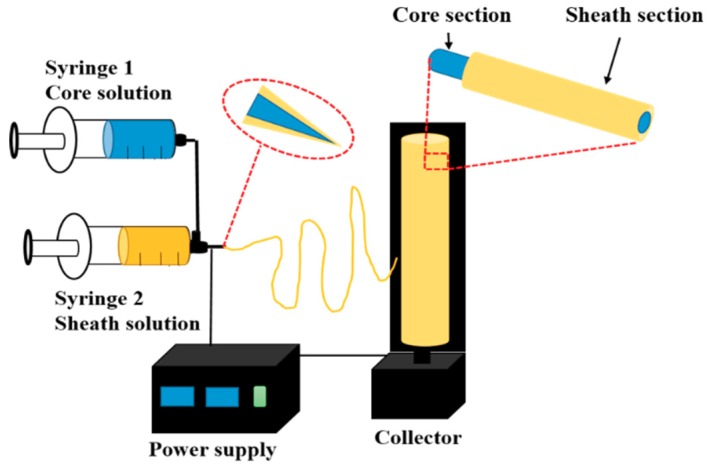
Schematic diagram showing the coaxial electrospinning system.

**Figure 4:**
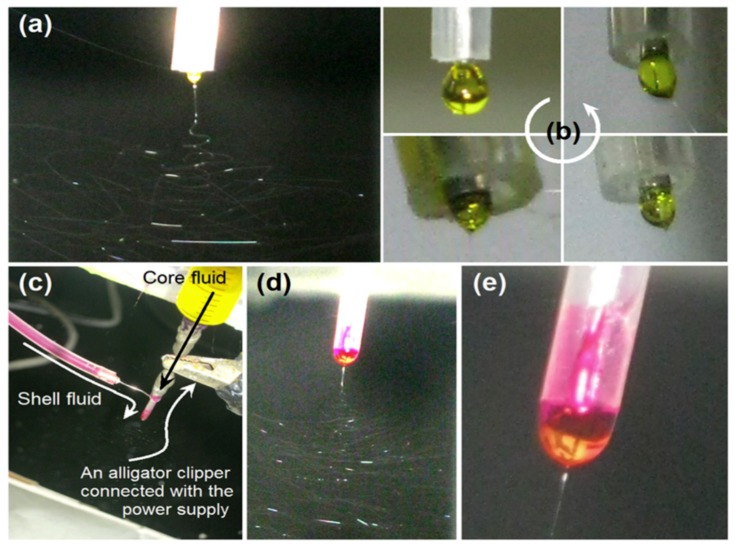
(**a**) Digital photographs showing a typical one-fluid electrospinning process, (**b**) formation of Taylor cone, (**c**) connection of spinneret in coaxial electrospinning, (**d**) enlarge image of the working region, and (**e**) Taylor cone with yellow core fluid encapsulate by rose-bengal shell solution [57].

The general setup and the fiber manufacturing process is conceptually similar to that of conventional electrospinning as mentioned in Section 1. In coaxial electrospinning, the spinneret is modified by inserting a smaller capillary to fit concentrically inside the bigger capillary in order to obtain a co-axial configuration (Figure 3). The outer needle contains a sheath solution whereas the inner one contains a core solution. The inner and outer nozzles pump two different spinning solutions simultaneously, producing a core-shell droplet at the exit of the nozzle. When the polymer solutions are charged with a high voltage, the accumulation of charge takes place predominantly on the surface of the sheath solution coming out of the coaxial capillary. The pendant droplet of the sheath solution elongates and stretches as a result of the charge–charge repulsion, forming a conical shape. When the charge accumulation reaches a threshold, a jet emerges from the tip of the deformed droplet directed towards the counter electrode. Finally, a core-shell fiber is deposited on the substrate [56]. Recently, Hai et al. [57] have reported the process of Taylor cone formation in typical one-fluid electrospinning and coaxial electrospinning processes; the digital photographic observations from their study are given in Figure 4.

### 3.3. Effects of Various Parameters on Coaxial Electrospinning

Like in ordinary electrospinning, the fiber morphology is governed by several factors. Therefore, the selection of appropriate solution parameters and processing conditions is very important for the steady generation of the core-sheath structure in coaxial electrospinning.

#### 3.3.1. Viscosities of the Solution

A good spinnability and sufficient viscosity of the sheath solution are required to produce enough viscous traction for the core solution. High viscosity can overcome the interfacial tension and form a stable Taylor cone. The core solution can have a lower viscosity (a minimum viscosity) to keep it intact and continuous inside the sheath fluid [55,58,59].

#### 3.3.2. Solution Concentration

The concentration of polymer solution is a key factor in electrospinning. If the polymer concentration is increased, the viscosity also increases. An increase in the concentrations of both core and sheath solutions results in a higher core-sheath fiber diameter. The diameters of the core or sheath fibers can be tuned by controlling the concentration of the core or sheath solutions [51].

#### 3.3.3. Solution Conductivities

In coaxial electrospinning, the sheath solution should be conductive whereas the core solution does not need to be conductive. By increasing the conductivity of the sheath solution, thinner fibers can be generated. Conversely, a highly conductive core solution can cause breakages due to the higher pulling rate in the core as compared to the sheath. A highly conductive sheath solution may affect the uniformity of the coaxial fibers due to the strong bending instability by abundant surface charges [55]. Hence, in order to obtain smooth nanofibers, the conductivity of core and sheath solutions should be maintained within an optimal range.

#### 3.3.4. Solvent/Solution Miscibility and Incompatibility

The interaction between the inner and outer solvents determines the miscibility of the two solutions; therefore, the choice of the solvents is very important in coaxial electrospinning [51]. The solvents in the core and sheath solution should be chosen in such a way that neither of them cause the precipitation of the other. Immiscible solutions are easily spinnable into the core-sheath forms due to the phase separation during electrospinning. The core and sheath solution with the same or miscible solvents are also capable of producing core-sheath nanofibers. The use of volatile solvents can form porous nanofibers; however, a too high volatility of the solvent may cause rapid evaporation leading to the formation of clogs of the spinneret. In this case, no core-sheath fiber can be obtained.

#### 3.3.5. Applied Voltage

For a solution, there is a small range of voltage within which a stable Taylor cone can be formed. If the applied voltage is too low, it may cause dripping of the two solutions forming a pendant drop at the exit of the nozzle. If the applied voltage is too high, it pulls the fluid jet inside the capillary and may form a clog [48,55]. Therefore, a good morphology of core-sheath nanofibers can be obtained by applying an optical range of the applied voltage.

#### 3.3.6. Solution Flow Rates

The flow rates regulate the amount of solution which exists in the concentric tip for electrospinning. An optimal flow rate ratio for the part of polymers is required. As compared to the flow rate of the sheath solution, the flow rate of the core solution plays an important role in preparing core-sheath nanofibers in coaxial electrospinning. At a very low flow rate of the core solution, the core phase became discontinuous which leads to the breakup of the core. On the other hand, a too high flow rate can cause pendent droplets to form. Generally, the flow rate of the core solution is lower than that of the sheath solution [51].

#### 3.3.7. Evaporation of Solvent

The solvent evaporation in coaxial electrospinning affects the Taylor cone formation and propagation and elongation of the jets. Rapid evaporation of solvents makes the solution dry at the nozzle tip before Taylor cone formation, whereas too slow evaporation causes the formation of drops [60]. The evaporation of solvents in both core and sheath solutions determines the morphology of the core and sheath structures. If the solvent in the shell solution evaporates at a higher rate than that of the core solution, the fibers collapse due to the atmospheric pressure. A high evaporation rate of the core solution also results in buckling and collapsed fibers due to the difference in pressure between the voids in the core and atmosphere.

#### 3.3.8. Tip-to-Collector Distance (TCD)

The tip to collector distance is also one of the important parameters in electrospinning. TCD is directly related to the flight time of the fluid jet [55]. If the distance is too short, it would likely result in the formation of an interconnected fiber mesh (or film) due to the limited time for the solvent to evaporate in the air. In cases where the distance is large, there is a higher chance for the solvent to evaporate. The higher evaporation of the solvent leads to thinner nanofiber formation.

#### 3.3.9. Nozzle Geometry

Since the nozzle geometry controls the flow rate, miscibility, and compatibility of the solutions, it has an influence on coaxial electrospinning. The diameter of the inner and outer nozzles and their geometry (length of core nozzle, the separation distance between the core and shell nozzles) may also influence the fiber morphology [60].

#### 3.3.10. Temperature and Humidity

In addition to the above parameters, coaxial electrospinning is also affected by atmospheric conditions such as temperature and humidity, etc.; however, these parameters have shown less influence (than the aforementioned parameters) on the formation and the uniformity of the coaxial fibers.

In summary, in order to prepare uniform and steady core-sheath nanofibers, optimal solution parameters should be determined. It can be seen that the parameters affecting the coaxial electrospinning are almost the same as in traditional single-fluid electrospinning, except the nozzle geometry and core-sheath fluid flow rates and ratio. Traditional coaxial electrospinning demands spinnable sheath fluid; however, modified coaxial electrospinning can generate core-sheath fibers by using unspinnable sheath fluids such as organic solvents [61,62]. In modified coaxial electrospinning, the sheath flow must match with the drawing process of the core fluid during spinning to obtain high quality core-sheath fibers. The sheath flow must match well with the drawing process of the core fluid during the spinning process [62]. Overall, the general features for coaxial electrospinning to produce the core-sheath fibers are as follows [51,55,60,62]:

(i) The sheath solution must be electrospinnable;
(ii) The viscosity of sheath solution should be relatively high compared to the core solution;
(iii) The viscosity of the core solution is required to be above the critical value, but should not be as high as the sheath solution;
(iv) A low surface tension of core solution;
(v) Sheath solution should be conductive;
(vi) In modified coaxial electrospinning, unspinnable sheath solutions can be used; however, the sheath flow must be adjusted well to match with the flow of the core fluid.

### 3.4. Core-Sheath Nanofibers for Drug Delivery Applications

The advancement of materials in drug delivery should be first attributed to the biocompatibility, which is a prerequisite for biomaterials [3,22,52]. In recent years, most studies have been concerned with synthetic or natural polymers in order to fabricate core-sheath nanofibers as effective drug carrier mediums. The degradation rate of the polymer plays an important role in drug release kinetics. The degradation behavior of the polymer may depend on several characteristics such as molecular weight, wettability, crystallinity, surface roughness, and the melting temperature of the polymer [52,63] Therefore, it is important to select a biocompatible polymeric material with an appropriate degradation rate to obtain the desired drug release kinetics. Table 2 presents some examples of core-sheath nanofibers by coaxial electrospinning for drug delivery applications. Some examples of widely used polymers and recent works related to drug release form core-sheath nanofibers by coaxial electrospinning are given below.

#### 3.4.1. Poly(vinyl alcohol) (PVA)

Water-soluble polymers offer additional advantages in electrospinning by eliminating the possible toxicity caused by solvents and represent a significant step towards clean and safe electrospinning. Among the various water-soluble polymers, poly(vinyl alcohol) (PVA), a synthetic polymer, is of great interest due to its desirable properties, such as its biocompatibility, adhesiveness, strength, swelling properties, non-carcinogenicity, film or fiber forming ability, etc. [64]. Recently, Yarin’s group [65] fabricated core-sheath nanofibers with PVA containing ciprofloxacin as a core and poly(methyl methacrylate) (PMMA) as a sheath layer in order to avoid the burst release of the drug. The nanofibers were explored for use in local drug delivery systems to treat periodontal disease and skin, bone, and joint infections. The study showed that the variation of flow rate ratios between core and shell during the spinning strongly affected nanofiber morphology and drug release. A lower amount of PVA in the core was helpful to prevent the burst release. In another study, Yarin [66] developed interesting mathematic models describing drug release from nanofibers.

Tiwari et al. [67] evaluated the usefulness of core-sheath fiber to minimize the burst release. In their study, the drug (metoclopramide hydrochloride) was loaded into the core (PVA) which was surrounded by different shells of various polymers (PCL, PLGA, and PLLA). The work clearly shows the sensitivity of the observed release to various parameters, related to both the process and material.

**Figure 5:**
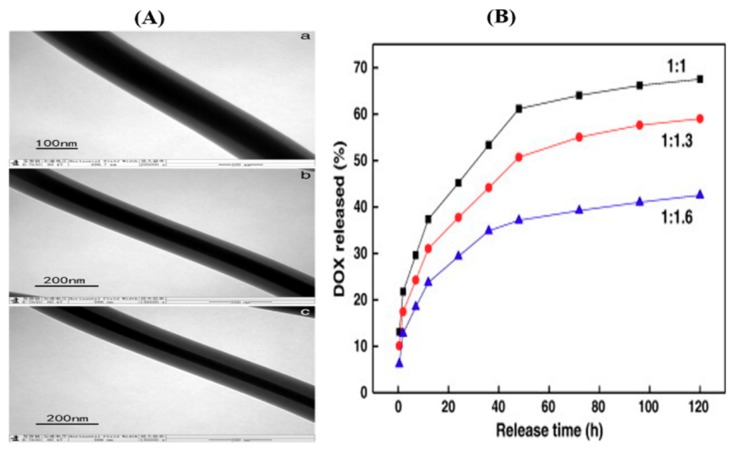
(**A**) TEM image of poly(vinyl alcohol)/chitosan (PVA/CS) core-sheath nanofibers with different feed ratios of (**a**) 1:1, (**b**) 1:1.3, and (**c**) 1:1.6 and (**B**) drug release profiles [68]. Reprinted with the permission from *Materials Science and Engineering: C*. Copyright Elsevier, 2014.

Yan et al. [68] used two water-soluble polymers, PVA and chitosan, as the core and sheath matrix, respectively, to prepare core-sheath fibers. Doxorubicin was used as a model drug and incorporated into the core matrix. PVA–chitosan nanofibers with different feed ratios were prepared as in Figure 5. The chitosan sheath was crosslinked by treatment with glutaraldehyde vapor to restrict the swelling of the polymer. The potential of nanofibers for use as a scaffold for chemotherapy of ovary cancer was evaluated in vitro against SKOV3 cancer cells and the obtained resulted indicated that the fibers were good in prohibiting cell attachment and proliferation. They observed that the release rate of drugs can be adjusted by changing the feed ratio and by keeping the feed ratio constant. More importantly, the core-sheath nanofibers exhibited controlled release of doxorubicin (DOX), showing potential for use in the chemotherapy of ovary cancer.

#### 3.4.2. Polycaprolactone (PCL)

PCL is a biocompatible and biodegradable synthetic polymer approved by the Food and Drug Administration (FDA). It has been widely used in drug delivery systems due to its broad range of molecular weight (from 3000 to 85,000 g/mol), solubility in varieties of solvents such as acetic acid, chloroform, methanol, benzene, dichloromethane, and its non-enzymatic degradation. PCL has been extensively explored in coaxial electrospinning for drug delivery applications.

Jing et al. [69] employed coaxial electrospinning in order to incorporate and control the release of two proteins, bovine serum albumin (BSA) and lysosome, from core-sheath nanofibers with protein containing polyethylene glycol (PEG) as a core and PCL as a shell. The protein release kinetics were characterized by a slight burst release during the first day followed by a relatively steady release extending over the complete time of the analysis of 30 days. The release profile of the incorporated proteins was found to be dependent on the feeding rate of the core solution. It was noticed that a higher feeding rate resulted in rapid protein release. In another study [70], the same group prepared biodegradable core-sheath fibers with PCL or a PCL/PEG blend as shell and BSA-dextran as core by coaxial electrospinning. PEG was added to the shell in order to further finely modulate the release behavior of BSA. Their study showed that the release rate of BSA increased with the PEG content in the shell.

He et al. [71] reported the fabrication of an anti-inflammatory agent loaded guided tissue regeneration membrane (GTRM) by coaxial electrospinning. In their study, metronidazole was loaded in PCL core fibers surrounded by gelatin as a sheath layer. They suggest that the dissolving of the gelatin sheath layer could be inhibited by crosslinking, and this resulted in sustained release of metronidazole for six days. The improved hydrophilicity also resulted in better cell adhesion and proliferation without toxicity. The prepared nanofiber membrane was capable of controlled drug release and inhibited the inflammatory response during the healing process, thereby showing better results as compared to PCL fibers only.

#### 3.4.3. Polyethylene oxide (PEO)

As a result of the solubility of hydrophilic drugs in the aqueous medium, it is challenging to maintain their prolonged release with a minimum release at the initial stage. Producing nanofibers from the blending solution of hydrophobic and hydrophilic polymers along with the drugs can reduce the burst release of hydrophilic drugs; however, the incompatibility between the drugs and hydrophilic polymers may cause the drug to migrate towards the surface of the nanofibers. In this regard, coaxial electrospinning can provide a better approach by encapsulating the drugs into the core portion of the nanofibers. The core-sheath nanofibers can be prepared by incorporating hydrophilic drugs with the polymer in the core and hydrophobic polymer as a sheath layer. Esmaeili and Haseli [72] prepared core-sheath and blend nanofibers to encapsulate tetracycline hydrochloride with PEO in the core and carboxymethyl cellulose in the sheath. As compared to the blend nanofibers, the core-sheath fibers showed a much slower and prolonged release of the drug. In the optimized core-sheath sample, the burst release was reduced from 54% to 26% and the total release was enhanced from 76% to 92% compared to the blend nanofibers. The initial burst release of the tetracycline hydrochloride in the case of the blend nanofibers might be associated with two factors: (i) The presence of the drugs on the surface of the blended nanofibers; and (ii) the hydrophilicity of the polymer which facilitates water uptake and swelling of the polymer matrix.

Recently, stimuli-responsive materials (smart materials) have gained extensive interest in controlled drug delivery applications. Various external stimuli such as light, temperature, magnetism, sound, etc., can bring change in the physical or chemical status of the smart materials. Li et al. [73] prepared a thermally switched drug delivery system, whose release can be tuned in response to a temperature change. Core-sheath fibers were prepared by coaxial electrospinning, in which drug encapsulated PEO forms a core layer, whereas a mixture of PCL and temperature stimuli-responsive nanogels form the sheath layer. The nanogels in the PCL matrix act as valves to generate a path for the diffusion of encapsulated drugs during the shrinkage and swelling above or under lower critical solution temperature (LCST) [73].

#### 3.4.4. Polyvinylpyrrolidone (PVP)

PVP is a water-soluble polymer with an amphiphilic character. As a result of its excellent properties, such as its solubility, film or fiber forming ability, adhesion and bonding, and biocompatibility, it has been extensively investigated for various applications in the biomedical field, including drug release. Core-sheath PVP/PCL nanofibers were developed by coaxial electrospinning in which graphene oxide (GO) sheets were blended into the core (PVP) solution to adjust the release behavior of vancomycin hydrochloride (VAN) [74]. The addition of GO into the nanofibers resulted in a reduction in burst release from 73% to 60%, and it was concluded that the amount of release can be tailored by adjusting the amount of GO in the core. The molecular interactions, such as hydrogen bonds, Var der Waal’s force, π–π bonds, between GO and the drugs played an important role in the typical biphasic release behavior the drug [74].

Yu et al. [75] prepared ketoprofen (KET) loaded core-sheath nanofibers by coaxial electrospinning using PVP as the sheath and ethyl cellulose (EC) as the core matrix and studied the drug release behavior. The drug was present in the polymer matrix in an amorphous state and the composite nanofibers provided a biphasic drug release profile consisting of an immediate and tunable sustained release. It was shown that the first and second phase of drug release could be tailored by adjusting the sheath flow rate and diffusion mechanism, respectively.

#### 3.4.5. Cellulose acetate (CA)

Cellulose is the primary structural component of the cell walls of green plants. It has been a material of choice in nanotechnology due to its advantageous properties including its biocompatibility, biodegradability, and regenerative properties [76,77]. Despite its advantageous properties, preparation of electrospun nanofibers is challenging because of its limited solubility in general organic solvents and disability to melt as a result of extra inter- and intramolecular bonding [31,78]. Cellulose acetate (CA), the acetate ester of cellulose, has been widely investigated for a wide variety of applications related to electrospun membranes [31]. The electrospun CA nanofibers can be converted into cellulose fibers by deacetylation or aqueous hydrolysis [79].

Yu et al. [76] applied the electrospinning process to develop ketoprofen (KET) loaded CA nanofibers. The drug was loaded into the CA fibers via both the single and modified coaxial electrospinning process. The core fluid was prepared by dissolving CA, KET, and a mixture of solvent (acetone, dimethylacetamide (DMAc), and ethanol), whereas the same mixed solvent was taken as sheath fluid. In addition, a CA solution containing KET in the same solvent was prepared for single fluid electrospinning. The nanofibers obtained from coaxial electrospinning had a smaller diameter, narrower size distribution, and smoother surface morphologies as compared to those generated from single fluid electrospinning. It was found that the fibers obtained from coaxial electrospinning offered a better zero-order drug release profile with a smaller tailing residue than that of single nozzle electrospinning. Another study by Yu’s group [80] showed a zero-order drug release profile from ketoprofen incorporated into CA nanofibers prepared via coaxial electrospinning using 2% CA solution as a sheath fluid and a mixture of CA, KET, and methylene blue in a mixed solvent system (acetone, DMAc, and ethanol) [76,80]. The obtained nanofibers provided zero-order drug release kinetics for 96 h via a diffusion mechanism.

#### 3.4.6. Zein

Zein, a mixture of proteins with different molecular weights in corn gluten meal, is widely known for its biocompatibility, biodegradability, antioxidant activity, and electrospinnability [81,82]. In recent years, it has drawn increasing attention in various applications, particularly in the biomedical field, including drug delivery [81,83,84,85,86,87]. In this regard, Huang et al. [84] investigated the preparation of ibuprofen (IBU) loaded fibers using a coaxial electrospinning process, in which zein/ibuprofen dissolved in ethanol aqueous solution and N,N-dimethylformamide were used as core and sheath fluids, respectively.

Jiang et al. [85] prepared core-sheath nanofibers from two polymers, PVP and Zein, to provide a biphasic drug release profile. Ketoprofen was exploited as the model drug in the study and was loaded into both the sheath and core fluids. Zein and PVA were selected as the core and sheath parts, respectively. Linear core-sheath nanofiber was produced with an average diameter of 730 ± 190 nm, in which the sheath part had a thickness of 90 nm. The study further showed good compatibility of the core and sheath matrix with KET due to hydrogen bonding. The dissolution tests showed an immediate release of 42.3% of the KET, followed by a sustained release over 10 h. Another study also demonstrated that the burst release can be prevented by using a blank or low concentration of zein solution in the sheath [88]. In another study, ferulic acid loaded zein was used as a core fluid and acetic acid was chosen as sheath fluid to stabilize the outer surface of the fibers [89]. The authors identified hydrogen bonding as a driving force of encapsulation of fluoric acid into the zein and the strategy of surface cross-linking was helpful for improving the release rate of the drug from the nanofibers [89].

## 4. Conclusions, Challenges, and Future Perspectives

In recent years, electrospinning technology has made a significant contribution in drug delivery applications. Optimization of the various parameters is important to design fibers with the desired morphology and function. Coaxial electrospinning ensures a homogeneous encapsulation of wide varieties of drugs into the core-sheath structured nanofiber along with a sustained release. Apart from drugs, several biomolecules have also been loaded into the core-sheath nanofibers via coaxial electrospinning. Additionally, some nonspinnable materials can be formed into a fiber structure due to the protection and guidance of the electrospinnable sheath layer. Easy loading of the drugs, mitigation of burst release, controlled sustained release, resemblance with the ECM, promotion of cell adhesion, and migration and proliferation are exciting features of core-sheath nanofibers which make them a suitable candidate in drug delivery applications.

It is evident from previous studies that core-sheath nanofibers are superior in terms of preserving the bioactivity of the biomolecules/drugs loaded in the fiber body and release behavior; however, there are several issues that must be addressed in order to obtain better results in drug delivery applications. To obtain the desired form of core-sheath nanofibers with satisfactory results, many experimental trails with different parameters are needed and this is complicated compared to conventional methods. The selection of core and sheath fluids is also important. The sheath layer may prevent rapid evaporation of the solvent during coaxial spinning; however, there is a chance that organic solvents will remain. To date, most reports of core-sheath fibers regarding drug delivery have focused on in vitro studies; therefore, in vivo studies with relevant preclinical studies are required for practical applications. Overall, coaxial electrospinning is an effective strategy for drug encapsulation and controlled release from nanofibers. Proper adjustment of parameters and a correct choice of core and sheath fluids are suggested to obtain good results, and further investigations and modifications are required to overcome the challenges in coaxial electrospinning.

Core-sheath devices should be further studied and advanced, in time making them more attractive for large-scale production. Furthermore, new strategies such as coaxial and tri-axial electrospinning combined with electrospraying can be adopted to prepare core-sheath fibers from spinnable and nonspinnable solutions [118,119]. In addition, designing a composite system using nanoparticles, hydrogels, and nanofibers may lead to significant advances in drug delivery applications. Recently, similar to the coaxial spinning technique, fabrication of drug loaded core-sheath nanostructures has been performed via coaxial and modified coaxial electrospraying processes [120,121]. The coaxial electrospraying method can secure drug entrapment into the core-sheath morphology, thereby giving the desired release kinetics [120]. Since both electrospinning and electrospraying are advanced nanofabrication methods that take advantage of the interactions between the working fluids and electrostatic energy, the contents and strategies reviewed in this paper as regards coaxial electrospinning and core-sheath nanofibers could also be applicable to coaxial electrospraying and core-shell nanoparticles.

## Figures and Tables

**Table 1:** Effect of various parameters on the properties of electrospun nanofibers.

Parameter	Effect	Reference
Applied voltage	High voltage generally reduces fiber diameter.	[14]
Concentration of solution	A higher concentration results in higher nanofiber diameter and the chances of bead formation are less. High concentration may clog the nozzle whereas low concentration may lead to sputtering.	[14,15,16]
Flow rate	Most flow rates are limited to 1 mL/h or lower. Increase in flow rate is associated with an increase in fiber diameter.	[16]
Inner diameter of needle	If large, beaded fiber may form.	[4]
Conductivity of solution	High conductivity leads to thinner nanofibers with less chances of bead formation.	[15]
Viscosity of solution	High viscosity leads to the formation of thicker and continuous nanofibers whereas low viscosity is associated with finer and shorter nanofibers.	[17]
Tip-to-collector distance (TCD)	Longer distance results in thinner fibers. If the distance is very short, nanofibers become sticky and tend to stick to each other, resulting in the formation of a film. Diameter also increases with the decrease in TCD.	[14]
Humidity	If humidity is high, beads and pores may form on nanofibers.	[18]
Volatility of the solvent	High volatility of the solvent is associated with higher chances of porosity and increased surface area.	[15]
Temperature	Both environmental and working fluid temperatures affect the fiber formation. Generally, the diameters of the nanofibers are uniform at higher temperatures.	[9]
Type of the collector	Smooth fibers can be obtained from metal collectors. Aligned fibers can be obtained using a conductive frame, rotating drum, or a wheel-like bobblin collector.	[19]

**Table 2:** Various core-sheath nanofibers for drug delivery applications.

Core Fluid	Sheath Fluid	Name of Drug	Application	Reference
PVA	PCL, PLLA PLGA	Metoclopramidehydrochloride	Drug delivery vehicle	[67]
PVA	PMMA	Ciprofloxacin	Periodontal disease and skin, bone, and joint infections	[65]
PVA	Chitosan	Doxorubicin	Chemotherapy against ovary cancer	[68]
Silk fibroin	PVA	Rosuvastatin	For enhancing osteogenesis of human adipose-derived stem cells	[90]
PCL	PCL	Ampicillin	Controlled release	[49]
PCL	PCL	Dipyridamole	Controlled release	[91]
PCL	Gelatin	Metronidazole	Controlled release	[71]
PCL	PEG	Salicylic acid	Studying the relationship between shell thickness and drug release rate	[42]
PCL	PCL	Ampicillin	Controlled release of a hydrophilic drug	[49]
Protein	PCL-PEG	BSA or PDGF	Controlled release of growth factor	[92]
Dextran	PCL, PLGA PLCL	Dextran	Controlled release of proteins and drugs for tissue engineering	[70,93,94]
PEG	PCL	BSA	Controlled release	[95]
pHMGCL, PVPD	PCL	rhTGF-β1	Sustained release of growth factor	[96]
PEO	PCL& PIPAAm/AAC-nanogels	MO	Thermally switched release	[73]
PEO	Carboxymethyl cellulose	Tetracycline hydrochloride	Drug delivery study	[72]
PEO	PCL-PEG	BMP-2	Drug release for bone tissue	[97]
PEO	PCL	FGF-2	Growth factor delivery for fibroblast proliferation	[98]
PEO	Eudragit S100	Indomethacin, mebeverine hydrochloride	Site specific drug release	[99]
PEG	PLA	Salicylic acid	Effect of pores in the drug release	[43]
PEG	PBSc	Triclosan/Curcumin	Drug release	[100]
PVP	CA	Amoxicillin	Hydrophilic drug release	[101]
PVP	EC	Maraviroc and Metronidazole	Drug release	[102]
PVP/GO	PCL	Vancomycin hydrochloride	Time-programmed biphasic drug release	[74]
PVP or PCL	PVP	Quercetin or Tamoxifen citrate	Dissolution of poorly water-soluble drugs	[103]
Ethyl cellulose	PVP	Ketoprofen	Drug release profile study	[75]
Naringin-loaded PVP	poly(lactic-co-glycolic acid)	Naringin Metronidazole	Fabrication of anti-infective guided tissue regeneration mats with promoting tissue regeneration	[104]
Zein	Acetic acid	Ferulic acid	Modified coaxial spinning. The effect of acetic acid to stabilize core fibers.	[89]
Zein	Zein	Ketoprofen	Hydrophobic drug release from protein fiber	[88]
Zein	PVP	Ketoprofen	Hydrophobic drug release from protein fiber	[85]
Tetracycline hydrochloride/Ethanol	Zein, PVA-SbQ	Tetracycline hydrochloride	Drug release study	[105]
PLGA	Collagen	Fibronectin and Cadherin 11	Dual drug delivery vehicle	[106]
PLLCL	Collagen	BMP2 Dexamethasone	Dual drug delivery vehicle	[107]
PLGA-HA	Collagen	Amoxicillin	Hydrophilic drug release from hydrophilic shell	[108]
Silk/collagen blend	Polyethylene oxide	Flurbiprofen and Vancomycin	Programmable release of anti-inflammatory and anti-bacterial agents	[35]
CA	CA	Ketoprofen	Drug release study	[80]
CA	Acetone-DMAc-ethanol	Ketoprofen	Controlled release	[76]
Sodiumhyaluronate	Cellulose acetate	Naproxen	Controlled release for wound dressing	[109]
Gelatin	PLLCL	Insulin, Hydrocortisone, and Retinoic acid	Dual drug delivery system Skin regeneration	[110]
PDLLA	PHB	Dimethyl oxalylglycine	Controlled release of hygroscopic drug	[111]
PMMA	Nylon	Ampicillin	Release of hydrophilic drug in hydrophobic solvent	[112]
PolyCD	PMAA	Proprannodol hydrochloride	Controlled release of hydrophobic drug	[113]
PLA	N-isopropylacrylamide	Combretastatin A4	Thermo-sensitivity study	[114]
Shellac	Ethanol/DMF	Ferulic acid	Colon targeted drug delivery	[115]
IBU solution in HFIP	Gliadin	Ibuprofen	Drug release behavior study	[116]
Gliadin	Gliadin	Ketoprofen	Drug release study	[117]
AAm/BIS-AAm	PLCL	BSA	Protein release	[59]

Poly(vinyl)alcohol (PVA), polycaprolactone (PCL), poly (lactic-co-glycolic acid) (PLGA), poly-L-lactic acid (PLLA), polymethyl(methacrylate) (PMMA), polyethylene glycol (PEG), polyethylene oxide (PEO), polylactic acid (PLA), cellulose acetate (CA), poly(L-lactide-co-caprolactone) (PLCL), poly(L-lactic acid)-co-poly(ϵ-caprolactone)(PLLCL), poly-d,l-lactic acid (PDLLA), polyhydroxybutyrate (PHB), poly-cyclodextrin (polyCD), graphene oxide (GO), platelet-derived growth factor (PDGF), bovine serum albumin (BSA), bone morphogenic protein (BMP), fibroblast growth factor (FGF), acrylamide (AAm), N,N’-methylene bisacrylamide (BIS-AAm), stilbazole quaternized (SbQ), recombinant human transforming growth factor (rh TGF-β1), methyl orange (MO).
